# New Books

**Published:** 2005-03

**Basic Cell Culture Protocols, 3rd ed.**

Cheryl D. Helgason, Cindy L. Miller

Totowa, NJ:Humana Press, 2004. 384 pp. ISBN: 1-58829-284-3, $125

**Cell Migration in Development and Disease**

Doris Wedlich

Hoboken, NJ:John Wiley & Sons, Inc., 2005. 450 pp. ISBN: 3-527-30587-4, $198

**Chemoinformatics in Drug Discovery**

Tudor I. Oprea, Ralmund Mannhold, Hugo Kubinyl, Gerd Folkers

Hoboken, NJ:John Wiley & Sons, Inc., 2005. 520 pp. ISBN: 3-527-30753-2, $185

**Children’s Health: International Historical Perspectives**

Cheryl Krasnick Warsh, Veronica Strong-Boag, eds.

Waterloo, Ontario:Wilfrid Laurier Press, 2005. 620 pp. ISBN: 0-88920-474-8, $38.95

**Comprehensive Handbook of Alcohol Related Pathology**

Victor Preedy, Ronald Watson, eds.

Burlington, MA:Elsevier, 2004. 2,192 pp. ISBN: 0-12-564370-5, $360

**Cytochrome P450: Structure, Mechanism, and Biochemistry**

Paul R. Ortiz de Montellano, ed.

New York:Springer-Verlag, 2005. 652 pp. ISBN: 0-306-48324-6, $149

**Encyclopedia of Biostatistics, 2nd ed.**

Peter Armitage, Theodore Colton

Hoboken, NJ:John Wiley & Sons, Inc., 2005. 6,100 pp. ISBN: 0-470-84907-X, $2995

**Environmental Policy, International Trade and Factor Markets**

C.C. Chao, E. Siu-Hung Yu

Burlington, MA:Elsevier, 2004. 252 pp. ISBN: 0-444-51708-1, $95

**Environmental Toxicology, Vol. 6**

T.P. Mommsen, T.W. Moon, eds.

Burlington, MA:Elsevier, 2005. 560 pp. ISBN: 0-444-51833-9, $260

**Global Sustainability: The Impact of Local Cultures, A New Perspective for Science and Engineering, Economics and Politics**

Peter A. Wilderer, Edward D. Schroeder, Horst Kopp

Hoboken, NJ:John Wiley & Sons, Inc., 2005. 266 pp. ISBN: 3-527-31236-6, $160

**Handbook of Toxicogenomics: Strategies and Applications**

Jurgen Borlak

Hoboken, NJ:John Wiley & Sons, Inc., 2005. 688 pp. ISBN: 3-527-30342-1, $195

**Health Implications of Perchlorate Ingestion**

Committee to Assess the Health Implications of Perchlorate Ingestion, National Research Council

Washington, DC:National Academies Press, 2005. 191 pp. ISBN: 0-309-09568-9, $42

**Preparative Chromatogography of Fine Chemical and Pharmaceutical Agents**

Henner Schmidt-Traub

Hoboken, NJ:John Wiley & Sons, Inc., 2005. 400 pp. ISBN: 3-527-30643-9, $145

**Preventing Childhood Obesity: Health in the Balance**

Jeffrey P. Koplan, Catharyn T. Liverman, Vivica A. Kraak, eds.

Washington, DC:National Academies Press, 2005. 434 pp. ISBN: 0-309-09196-9, $44.95

**Proceedings of the 3rd Asia-Pacific Bioinformatics Conference**

Yi-Ping Phoebe Chen, Limsoon Wong

Hackensack, NJ:World Scientific, 2005. 400 pp. ISBN: 1-86094-477-9, $78

**Proteins: Structure and Function**

David Whitford

Hoboken, NJ:John Wiley & Sons, Inc., 2005. 600 pp. ISBN: 0-471-49894-7, $79.95

**Statistical Methods in Bioinformatics An Introduction, 2nd ed.**

Warren J. Ewens, Gregory Grant

New York:Springer-Verlag, 2004. 588 pp. ISBN: 0-387-40082-6, $89.95

**Statistics for Experimenters: Design, Innovation, and Discovery, 2nd ed.**

George E.P. Box, J. Stuart Hunter

Hoboken, NJ:John Wiley & Sons, Inc., 2005. 736 pp. ISBN: 0-471-71813-0, $99.95

**Systems Biology in Practice: Concepts, Implementation and Application**

Edda Klipp, Ralf Herwig, Axel Kowald, Christoph Wierling, Hans Lehrach

Hoboken, NJ:John Wiley & Sons, Inc., 2005. 300 pp. ISBN: 3-527-31078-9, $120

